# Uncovering the role of Rad51 in homologous recombination-mediated antigenic diversification in the human malaria parasite *Plasmodium falciparum*

**DOI:** 10.3389/fmolb.2023.1223682

**Published:** 2023-08-01

**Authors:** Pratap Vydyam, Nabamita Roy, Mrinal Kanti Bhattacharyya

**Affiliations:** Department of Biochemistry, School of Life Sciences, University of Hyderabad, Hyderabad, India

**Keywords:** malaria, *Plasmodium falciparum*, homeologous recombination, *var* gene rearrangement, antigenic variation, PfRad51

## Abstract

The human malaria parasite *Plasmodium falciparum* maintains the chronicity of infections through antigenic variation, a well-coordinated immune evasion mechanism. The most prominent molecular determinant of antigenic variation in this parasite includes the members of the *var* multigene family. Homologous recombination (HR)-mediated genomic rearrangements have been implicated to play a major role in *var* gene diversification. However, the key molecular factors involved in the generation of diversity at *var* loci are less known. Here, we tested the hypothesis that PfRad51 could carry out recombination between *var* genes that are not homologous but homeologous in nature. We employed the whole-genome sequencing (WGS) approach to investigate recombination events among *var* sequences over 100 generations and compared the rate of sequence rearrangement at the *var* loci in both PfRad51-proficient and -deficient parasite lines. This brief report provides evidence that the loss of the key recombinase function renders the parasite with inefficient HR and results in fewer recombination events among the *var* sequences, thereby impacting the diversification of the *var* gene repertoire.

## Introduction

Malaria is one of the leading life-threatening problems worldwide. This severity is attributed to chronic infections and the innate immune evasion strategy possessed by *Plasmodium falciparum*. One of the remarkable biological features of *P. falciparum* is the expression of polymorphic family proteins on the surface of infected erythrocytes (IE) called *P. falciparum* erythrocyte membrane protein 1 (*Pf*EMP1), a major antigenic determinant that enables IE binding and evades the host antibody response by clonal antigenic variation (Kyes et al., 2001). Antigenic diversity generated by the polymorphic, *var* multigene family is the foundation for *Plasmodium* immune evasion (Scherf et al., 2008; Petter and Duffy, 2015). *Pf*EMP1 is encoded by a family of hypervariable genes with long ORFs, namely, *var* genes. In *Plasmodium*, nearly 60 copies of *var* genes per haploid genome are clustered majorly at the sub-telomeric region, with a diverse structure, having a large exon-1 (4–10 kb) that encodes a variable extracellular domain and a small conserved exon-2 encoding the cytosolic domain of *Pf*EMP1 which facilitates anchoring to the host erythrocyte membrane (Baruch et al., 1995; Su et al., 1995; Gardner et al., 2002). At the subdomain level, there are homology blocks within the *Pf*EMP1 encoding region that are separated by hypervariable regions, which presumably facilitates recombination between semi-homologous genes (Bull et al., 2008). The lesser degree of sequence conservation in the hypervariable *var* loci, in comparison to the rest of the genome, suggests that they must undergo a high rate of mutation or recombination (Bockhorst et al., 2007). The majority of recombination events take place within or near *var* genes, and these are counted as recombination hotspots (Taylor et al., 2000; Mu et al., 2005; Mu et al., 2007). During asexual replication of parasites, these *var* genes undergo gene recombination through mitosis, resulting in diversification. An increase in diversity is achieved when ectopic (non-allelic) recombination events take place between *var* genes (Duffy et al., 2009). The frequency of mitotic recombination and the generation of novel *var* sequences were determined through whole-genome sequencing and microarray analyses (Bopp et al., 2013; Claessens et al., 2014). A high frequency of *var* gene rearrangements was observed in the asexual stage of parasites (Claessens et al., 2014). In the progeny generated from an experimental genetic cross between two different strains of *Plasmodium*, it was observed that the frequency of *var* recombination is much higher than the overall meiotic recombination rate for the bulk of the genome (Freitas-Junior et al., 2000). Mitotic recombination during asexual replication is thought to stem from the necessity of repairing DNA double-strand breaks that are spontaneously generated during the normal course of the development of the parasite (Kolodner et al., 2002; Moynahan and Jasin, 2010). Apparently, in *P. falciparum*, DSB repair is considered to rely almost exclusively on homologous recombination (HR) due to a lack of efficient classical non-homologous end joining (NHEJ) (Kirkman and Deitsch, 2012; Kirkman et al., 2014). The genome of *Plasmodium* is haploid in most of its life cycle (except for a short period during the mosquito stage). Hence, the unbroken sister chromatid acts as homologous templates for the HR mechanism. However, for the repair of the genes belonging to the multigene family, several additional homologous templates are available. Such a recombination event potentially gives rise to the generation of new mosaic sequences.

During DSB repair, strand exchange protein Rad51 utilises the unbroken homologous DNA template, such as the sister chromatid or homologous chromosome, to repair the broken chromosome. Rad51 can distinguish the homologous template from the non-homologous template during this process. However, the stringency of such discrimination is not complete. For example, Rad51 could carry out the strand-exchange reaction between the maternal allele and the paternal allele during meiotic recombination. Thus, Rad51 can tolerate and bypass short stretches of heterologous sequences that are present in the long stretch of homologous sequences. Such templates that contain short blocks of heterologous sequences in the homologous templates are called homeologous sequences. In the model eukaryote *Saccharomyces cerevisiae*, Rad51 could bypass 6–9 bp heterologous blocks and in *E. coli*, RecA could bypass up to 200-bp-long heterologous blocks (Iype et al., 1994).

In *P. falciparum*, the recombinase orthologue PfRad51 was identified, and its strand-exchange activity was characterised (Bhattacharyya and Kumar, 2003; Bhattacharyya et al., 2005). A chemical inhibitor of PfRad51 was found to block the ATPase activity, strand-exchange activity, and multimerization of the PfRad51 protein (Vydyam et al., 2019). In a genetic study, the overexpression of a dominant negative mutant of PfRad51^K143R^ abrogated DSB repair in *Plasmodium berghei*, emphasising the fact that HR is the major DSB repair mechanism in this parasite. Rad51 functions as a hexameric protein, with each monomer requiring ATPase activity. The dominant negative mutant PfRad51^K143R^ has the ability to interact within the hexameric ring along with wild-type proteins, effectively suppressing the activity of the wild-type Rad51 protein. Thus, this mutant exerts a dominant negative effect, inhibiting the function of Rad51 (Roy et al., 2014).

The following two questions remain unanswered: whether the PfRad51-mediated HR pathway actually contributes to *var* gene rearrangement? Whether the role of PfRad51 could be compensated by another HR protein? In this study, aiming at answering the aforementioned questions, we used a transgenic parasite strain harbouring a dominant negative allele of PfRad51. Initially, we determined the frequencies and length of heterologous blocks within *var* sequences. We generated dominant negative *Pf*rad51 (*Pf*Rad51^K143R^) parasite lines and monitored the rate of rearrangements over hundred generations for both PfRad51^wild-type^ and mutant parasite lines. Whole-genome sequencing analyses revealed a significant decrease in the rate of change of nucleotide variations (NVs) and structural variations (SVs) at *var* genes in PfRad51 mutant parasites, suggesting a potential role in *var* gene diversity.

## Materials and methods

### Bioinformatics analysis of heterologous blocks in *var* genes

To study the nature of heterology blocks, we obtained the genomic DNA sequence from the *Plasmodium* genome database, PlasmoDB. We retrieved the *var* gene sequences and grouped them according to their upstream promoter sequence (*UPS*) region. Sequence alignment was carried out to get the heterologous blocks among *var* genes using the bioinformatics tool CLUSTALW. We analysed two types of heterologous blocks: nucleotide substitution and insertion/deletion. The length and the occurrence frequencies of the heterologous blocks were plotted using GraphPad Prism.

### Generation of the HR-deficient (*Pf*Rad51^K143R^) parasite line

Synchronous early ring-stage parasites with 6%–8% parasitaemia resuspended in pre-warmed Cytomix buffer (10 mM K_2_HPO_4_ pH 7.6, 120 mM KCl, 0.15 mM CaCl_2_, 25 mM HEPES pH 7.6, 2 mM EGTA pH 7.6, and 5 mM MgCl_2_) were electroporated with 100 µg plasmid DNA (*Pf*CENV3–*Pf*Rad51^K143R^) using a Bio-Rad Gene Pulsar (0.31 kV, 950 µF, and ∝ resistance). Post-transfection, once the parasitaemia reached 4%–8%, the cells were kept under blasticidine (Sigma-Aldrich) pressure (2.4 μg/mL) until the transfectants appeared. The presence of the plasmid *Pf*CENV3–*Pf*Rad51^K143R^ was confirmed by amplifying the PCR fragment size of 1,325 bp using primer pairs OMKB 198 (GGT GAG CTA GCT AAT AGG C) and OMKB 462 (TTA CAC TTT ATG CTT CCG GC). Similarly, the transfectants possessing the empty *Pf*CENV3 vector were confirmed with vector-specific primers OMKB 501 (ACA ACC ATT ACC TGT CCA C) and OMKB 462 that yielded an amplicon of 1,171 bp.

### Genomic DNA isolation from *P. falciparum 3D7 in vitro* cultures

The parasite culture with 6%–8% parasitaemia was harvested and treated with saponin (0.15%) (Sigma-Aldrich) at 37°C for 30 min. The parasite pellet was resuspended in the lysis buffer (10 mM Tris-HCl pH 8, 20 mM EDTA pH 8, 0.5% SDS, and 0.1 mg proteinase K) and incubated for 3 h with intermittent mixing. After incubation, equal volumes of PCIA [phenol, chloroform, and isoamyl alcohol at a ratio of 25:24:1 (v/v/v)] were added, and the aqueous layer was collected upon centrifugation at 12,000 rpm for 15 min at room temperature. The aqueous layer was treated with RNase for 30 min at 37°C, followed by PCIA treatment. The sample was subjected to precipitation by addition of sodium acetate (1/10th vol) and 100% ethanol (2.2 vol) and incubated at −80°C overnight, followed by centrifugation at 12,000 rpm at 4°C, and the DNA were resuspended in 1x TE buffer.

### Isogenic strain generation by limiting dilution

The previously published protocol was followed for cloning and re-cloning parasite lines using the limiting dilution method (Butterworth et al., 2011). Briefly, parasite strains of *Pf*3D7, *Pf*CENV3, and *Pf*DNRad51 were cultured routinely *in vitro*. All isogenic lines were subjected to re-cloning at their designated time points, including the 0th, 50th, and 100th generation. It was made sure that the culture had >90% of the singly infected erythrocytes. Parasite cultures were seeded at 0.2 to 0.5 parasites per well in a 96-well plate in 100 µL with 2% HCT Complete medium. The culture media were changed on days 4, 7, 11, 14, and 18. Sufficient number of parasites was visible after 21 days, and the culture was utilised for further experiments. It is worth mentioning that each re-cloning process, from the time of seeding to the time of harvesting parasites for DNA isolation, took approximately 10 generations time. The *N*th generation is defined as the day of seeding and not the day of harvesting parasites.

### Preparation of DNA for whole-genome sequencing

A measure of 2 μg of genomic DNA samples from each time point was used for whole-genome shotgun (WGS) library preparation with a fragment length ranging from 300 to 500 bp. The generated DNA library was subjected to paired-end sequencing of 150 bp using the Illumina NextSeq (150 × 2) sequencing platform (Genotypic Technology, Bangalore, India). The WGS analysis entailed the sequencing of the 23-MB *Plasmodium falciparum* genome using 150-bp reads, which resulted in a genome coverage of ×20. This sequencing approach yielded an adequate quantity of high-quality reads, exceeding 2 million reads per clone for both the wild-type and mutant parasite lines. The extensive dataset provided a comprehensive perspective of the genomic landscape, facilitating a thorough examination of the genetic composition and variations. These high-quality reads were aligned with the reference genome sequence of *Pf3D7* (PlasmoDB.), with the coverage of each sample at a depth of ×10 and ×20. We identified both nucleotide variations that include single-nucleotide polymorphisms (SNPs) and insertions/deletions (INDELs) between 1 and 20 bp size and structural variations (insertions, deletions, translocations, and inversions with the size of >300 bp) using SAM, BCF, and DELLY tools.

### Generation of Circos plots

To visualise the WGS data for the easy identification of similarities and differences between genomic sequences, we prepared Circos plots for each sample using an online tool Circa (http://circos.ca). The circular ideogram layout facilitates the display of all chromosomes and the position of SNPs, INDELs, and structural variations on each chromosome. The data were represented with identified SNPs, INDELs, and translocations of six different samples from both wild-type and *Pf*Rad51 mutant parasites in six different plots. A typical Circos plot contained all 14 chromosomes of *P. falciparum* arranged in an ideogram track. Both SNPs and INDELs in the core genome and *var* genes are represented as a histogram and Scatchard plot, respectively. The translocations are represented in blue and orange lines for the core genome and *var* genes, respectively.

### Estimation of the rate of change of nucleotide and structural variation calculation at *var* genes and core genome

We estimated the rate of change of both nucleotide variations (SNPs and INDELs) and structural variations per generation per nucleotide in wild-type and *Pf*Rad51 mutant parasites. The rate of change was calculated by taking the total number of unique changes in nucleotide or structural variations present in that particular strain per generation per nucleotide. The genome sequences of the 0th generation of both wild-type and *Pf*Rad51 mutant parasites were taken as the reference, and any new or missing nucleotide variations or structural variations were taken as changes. The rate of change for the *var* gene region was calculated by considering the total size of the *var* genes, while the rate of change for the rest of the genome was calculated considering the genome size.

## Results

### Identification and distribution of heterologous blocks in *Plasmodium var* genes

**FIGURE 1:**
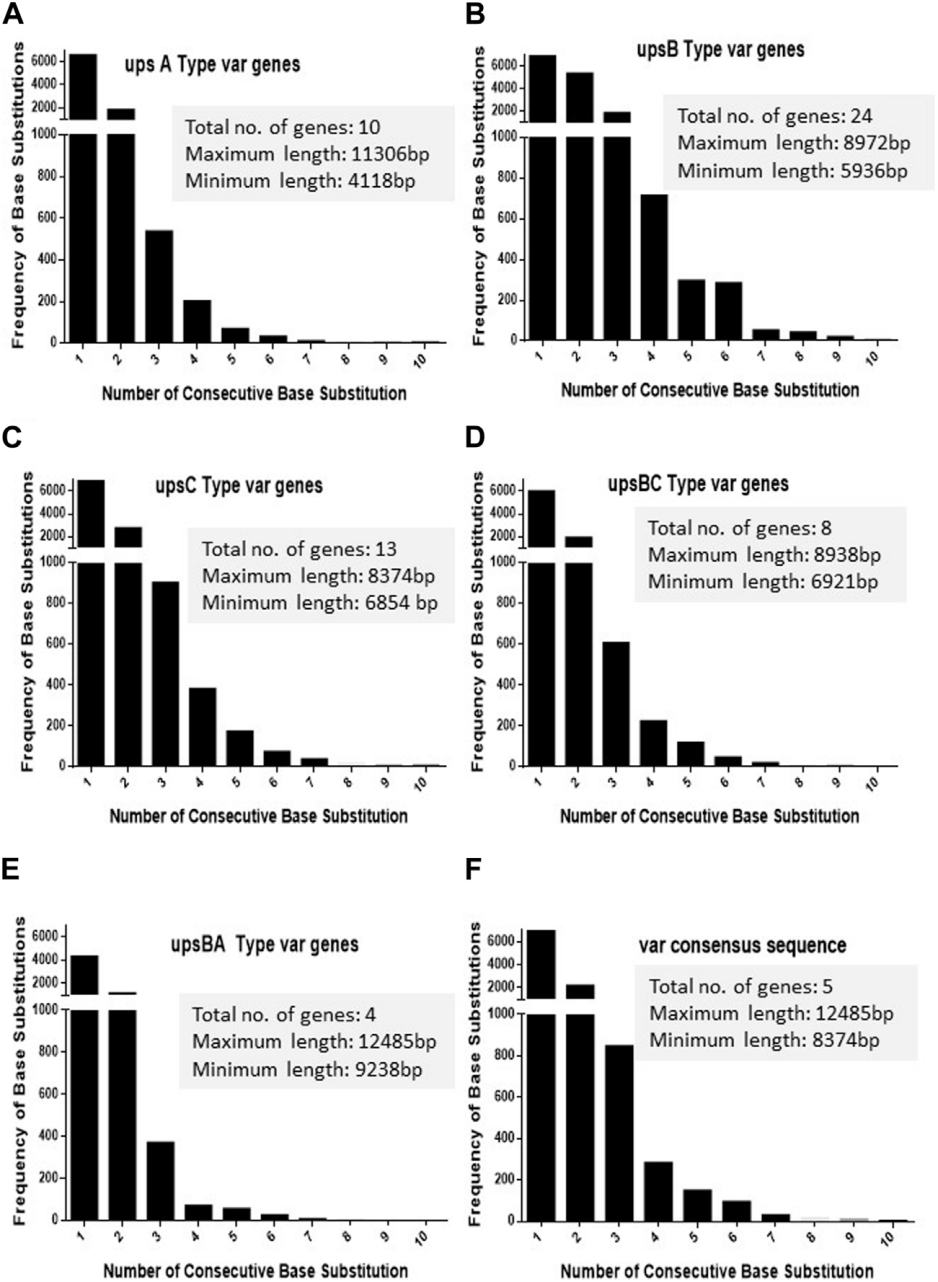
Distribution of heterologous blocks due to consecutive base substitutions in *var* genes. The *var* genes were divided into five groups according to their *UPS* type. **(A–E)** The number of consecutive base substitutions were determined and plotted against the frequency of their occurrence. The total number of genes in each group, along with the maximum and minimum lengths of the genes is mentioned in the box. **(F)** The consensus *var* sequences from each *UPS* group were compared against each other, and the frequencies of consecutive base substitutions were plotted.

**FIGURE 2:**
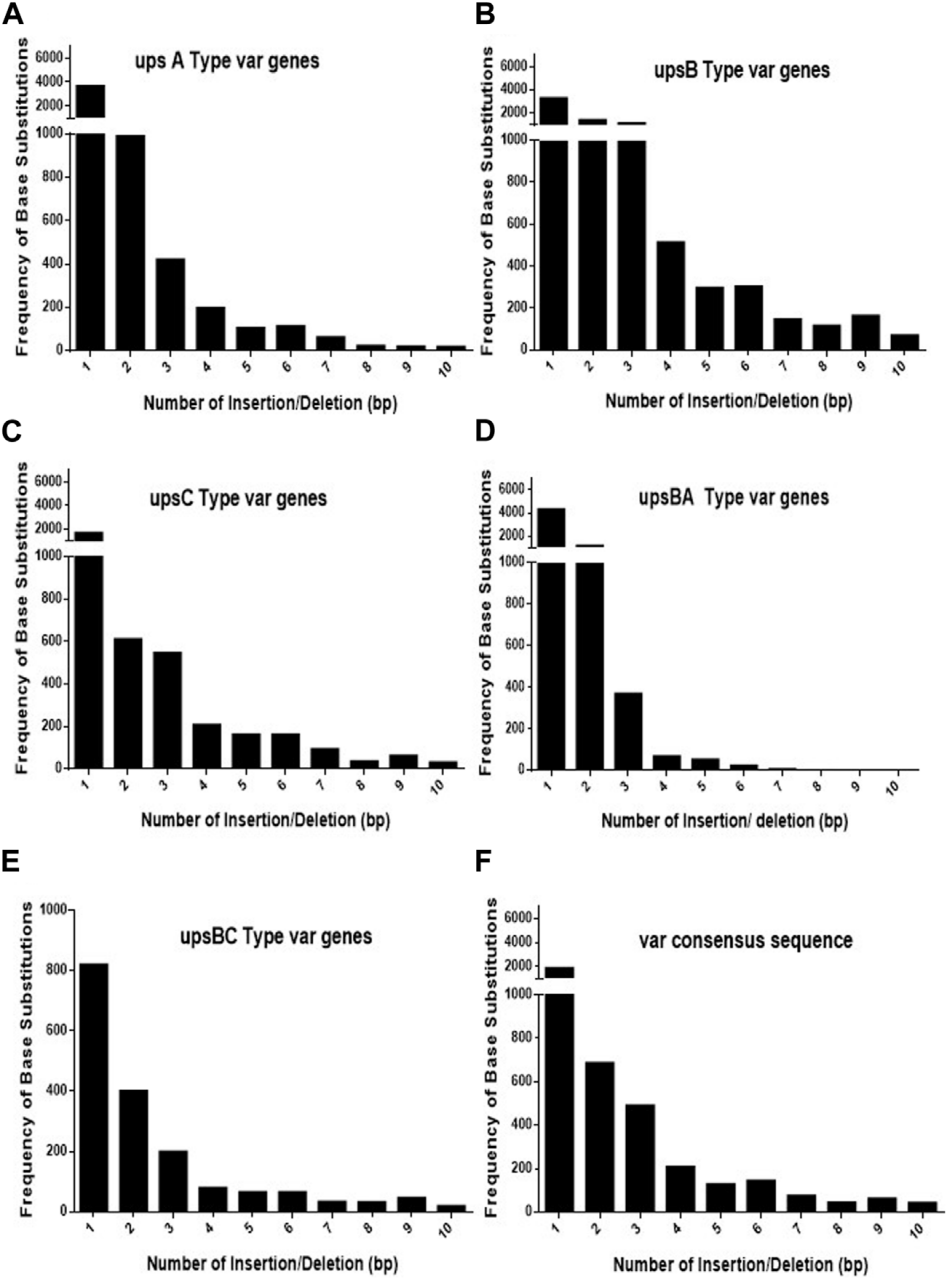
Distribution of heterologous blocks due to consecutive insertions or deletions of bases in *var* genes. **(A–E)** The *var* genes grouped into five *UPS* groups were inspected for the number of consecutive insertions/deletions of bases and plotted against the frequency of their occurrence. **(F)** The consensus *var* sequences from each *UPS* group were compared against each other, and the frequencies of the consecutive INDELs were plotted.

As *var* sequences are homeologous in nature with respect to each other, it is important to estimate the length and the frequency of such heterologous blocks at *var* genes. It is intitutive that shorter heterologous blocks could be bypassed during recombination events between *var* sequences, while the longer blocks may not be tolerated. To analyse *var* genes, we retrieved *var* gene sequences (*P. falciparum 3D7* genome) from the PlasmoDB database. Sequence alignment was carried out for each *UPS* group of *var* genes using CLUSTALW (DNASTAR software), and the length of the heterologous blocks was analysed. After the alignment of the sequence, deletions/insertions or substitutions of the base pair that caused sequence divergence were identified as heterologous blocks. Every single-base substitution from each *UPS* group was analysed and represented in graphs by taking the length of heterologous blocks and the total number within the group (Figures 1A–E). The consensus sequences from each *UPS* group were also analysed to determine the lengths and frequencies of heterologous blocks across *UPS* groups (Figure 1F). Similarly, insertions/deletions from each *UPS* group and across UPS groups were also analysed (Figures 2A–F). From the data, it is evident that *var* genes mostly have short heterologous blocks, ranging from 1 to 6 bp. Additionally, the length of such heterologous blocks was inversely proportional to the frequency of their occurrence. There are very few base substitution blocks with lengths greater than 6 bps, and a similar pattern was also observed with insertions/deletions.

### Parasites with inefficient HR manifest a reduction in the rate of change of nucleotide and structural variations in the genome

**FIGURE 3:**
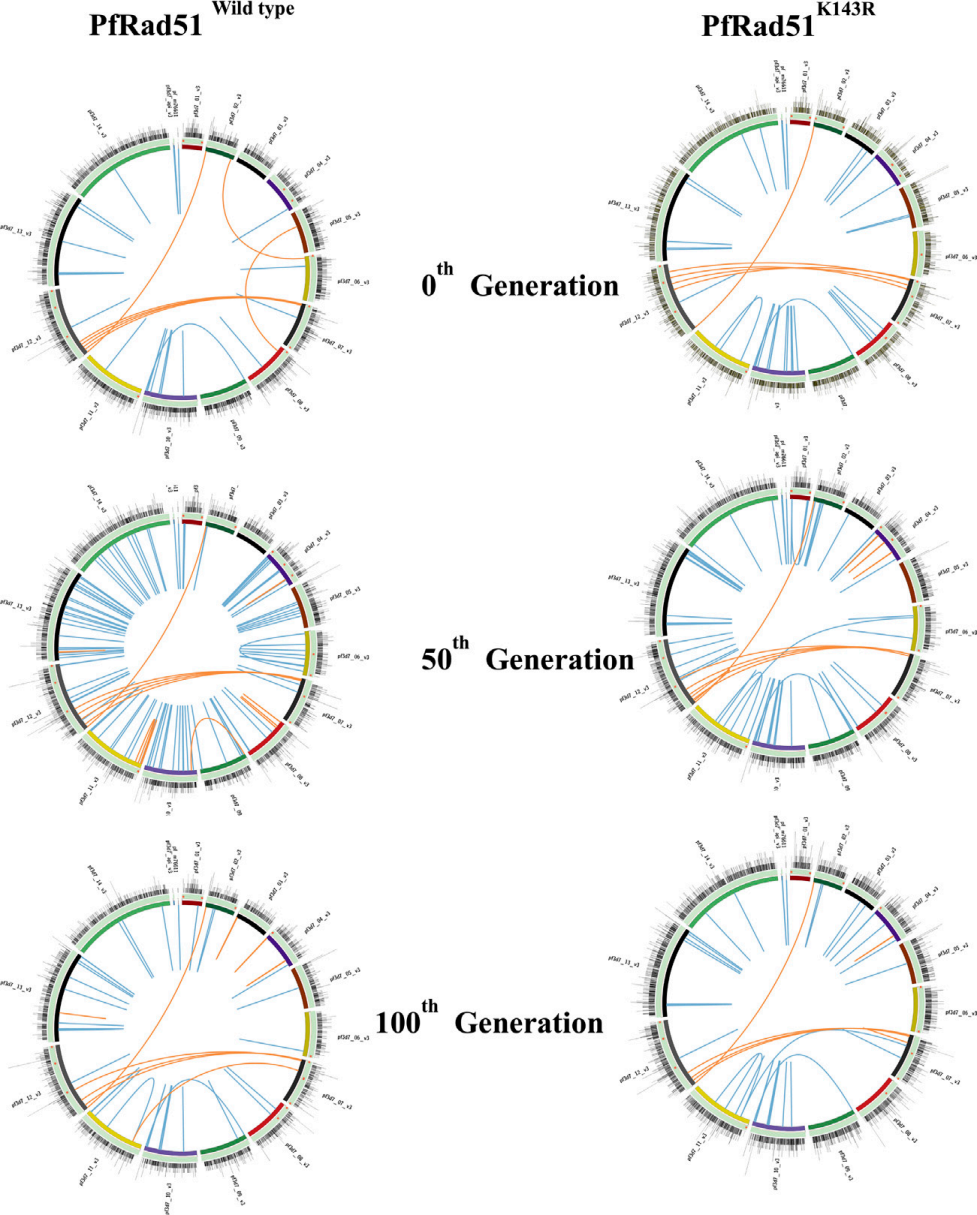
Visualisation of genomic variations on chromosomes of both wild-type and HR-deficient parasites. Each Circos plot illustrated here is the representative image of the respective generation of both wild-type (PfRad51^wild-type^) and HR-deficient (PfRad51^K143R^) parasite lines. All chromosomes in each Circos are represented as arcs in a circle, arranged in the different coloured ideogram. The outer most track with nucleotide variations (SNPs and INDELs) scattered in the genome is represented as histograms. The Scatchard plot in each track represents NVs at *var* genes (orange dots). Structural variations (translocations, inversions, deletions, and duplications >300 bp) are depicted at the innermost track of the core genome (blue lines) and *var* gene region (orange lines).

Since the lengths of heterologous blocks were found to be below the permissible limit of heterology bypass by eukaryotic Rad51, we hypothesised that PfRad51 should be able to carry out recombination between homeologous var sequences. To investigate the role of PfRad51 in *var* gene recombination, we have expressed a dominant negative allele of PfRad51^K143R^ in the *P. falciparum in vitro* culture. This particular mutant was reported to be defective in DSB repair (Jha et al., 2021). We have chosen a *Pf*3D7 strain for our investigation of gene diversification studies because of its complete annotated genome (Gardner et al., 2002). We sought to identify nucleotide and structural variations at the *var* loci and in the core genome. To this end, we successively cultured isogenic lines of both wild-type and mutant parasites for 100 generations. At designated time intervals (0th, 50th, and 100th generation), we re-cloned both parasite lines and isolate genomic DNA from individual clones, and representative clones were subjected to Illumina platform-based WGS to obtain the mutation score as described in methodology. The total number of nucleotide variants (SNPs and micro-INDELs with the size of <20 bp) and structural variations (translocations, inversions, and duplications with >300 bp) distributed throughout the genome were identified at all the time points of both parasite lines using SAM, DELLY, and BCF tools (Morel et al., 1994; Holmes et al., 2001). A detailed inspection of NVs generated in both wild-type and DN parasites were represented as a histogram on all 14 chromosomes in Circos plots (Figure 3). Similarly, structural variations are represented as blue and orange lines in Circos maps of the respective generation for the core genome and *var* gene region, respectively, of both parasite lines (Figure 3).

**FIGURE 4:**
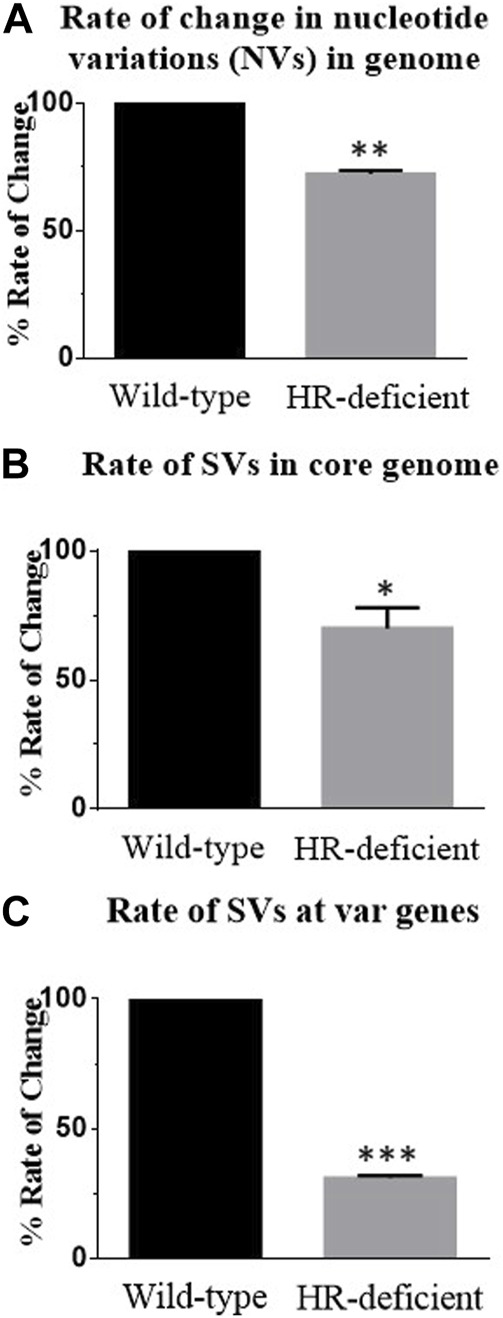
Reduced rate of change of nucleotide variations and structural variations at both the core genome and *var* genes in the HR-deficient parasite line. **(A)** Bar diagram showing the rate of change of nucleotide variations observed in the entire genome of wild-type and HR-deficient parasites. Total unique NVs present in the strain/per generations/genome size gives the rate of change in NVs. **(B)** Bar diagram showing the rate of change of structural variations (SVs) observed in the entire genome of both wild-type and HR-deficient parasites. Total unique SVs observed in the strain/number of generations/genome size give the rate of change in SVs. **(C)** The graph represents the rate of change of SVs in *var* genes calculated by taking the unique SVs generated only in *var* genes/generations/base pairs. The mean value ± SEM is plotted. Student’s *t*-tests were performed for determining the significance. Asterisks indicate values significantly different from the control, given as follows: ***p <0.001, **p <0.01, and *p <0.05.

To compare the rate of rearrangement between wild-type and mutant parasite lines, we scored the unique change and calculated the rate of change in nucleotide and structural variations. We carried out detailed inspections of NVs and SVs generated in both wild-type and DN parasites. By taking the 0th generation as a reference, the appearance or disappearance of any variation was considered a single change. We scored all such novel and missing NVs or SVs from WGS of both parasite lines in subsequent generations using BCF tools. Taking the 0th generation as the reference point, we estimated the genome-wide rate of change of NVs and SVs per generation per nucleotide in both parasite lines and observed nearly a 30% reduction in the rate of change in HR-deficient parasites (Figures 4A, B).

To gain insights into recombination events at the *var* loci, we estimated the rate of change of SVs at *var* genes. After normalisation with the size of the core genome, the total number of unique SVs was found to be more at the *var* region than the core genome, and it was found that in HR-deficient parasites, nearly a 70% reduction in the rate of change at *var* genes was observed (Figure 4C). Together these results suggest that PfRad51-deficient parasites undergo fewer recombination events and thereby decrease the rate of change. In conclusion, the lack of a functional *Pf*Rad51 led to a significant decrease in the rate of *var* gene diversity in *Plasmodium*.

## Discussion

Here, we provide evidence that the homologous recombination efficiency is positively correlated with *var* gene recombination in *P. falciparum*. Our results corroborate well with a previous finding in which a link between DSB repair by the HR mechanism and a rapid diversification of the sub-telomeric *var* repertoire was revealed (Zhang et al., 2019). We also revealed that the functional loss of the key recombinase PfRad51 severely compromises the rate of *var* gene recombination. A previous study demonstrated that RecQ helicase PfBLM, another important protein of the *Plasmodium* HR pathway, plays a negative regulatory role in *var* gene recombination (Claessens et al., 2018). Thus, the interplay between *Plasmodium* HR proteins appears to regulate the outcome of *var* gene recombination.

In the absolute sense, if the PfRad51-mediated HR mechanism is the only means for generating *var* gene diversification, then one would expect a complete reduction in the rate of rearrangement in the PfRad51 loss-of-function mutant parasite line. Here, we observed a marked decrease in the rate, but *var* rearrangements were not abrogated. This could be explained by the fact that we did not conduct the experiment on a PfRad51 null mutant; instead, we overexpressed a dominant negative allele of PfRad51^K143R^, which does not allow the PfRad51^wild-type^ protein to function. As PfRad51 works as a multimeric protein, the inclusion of even one molecule of the mutant protein in the multimeric complex could inactivate its function (Roy et al., 2014). However, there is always a possibility that a certain multimeric PfRad51 complex may consist of all the PfRad51^wild-type^ monomers, and such a complex will be functionally active and capable of *var* rearrangements. Another possibility is that the absence of PfRad51 can be compensated by the Rad55–Rad57 complex. Indeed, in the *P. falciparum* genome, a putative Rad57-like protein has been identified (PF3D7_0419300). However, a Rad55-like protein seems to be absent in *P. falciparum*. A third possibility could be that the loss of HR activity could be compensated by the less frequent alt-NHEJ mechanism (Kirkman et al., 2014). This possibility seems very unlikely, as the loss of HR could not be compensated by any other mechanism for methyl methane sulfonate (MMS)-induced DSB repair (Roy et al., 2014). Nonetheless, our finding suggests that PfRad51-mediated HR plays a major role in *var* recombination.

**FIGURE 5:**
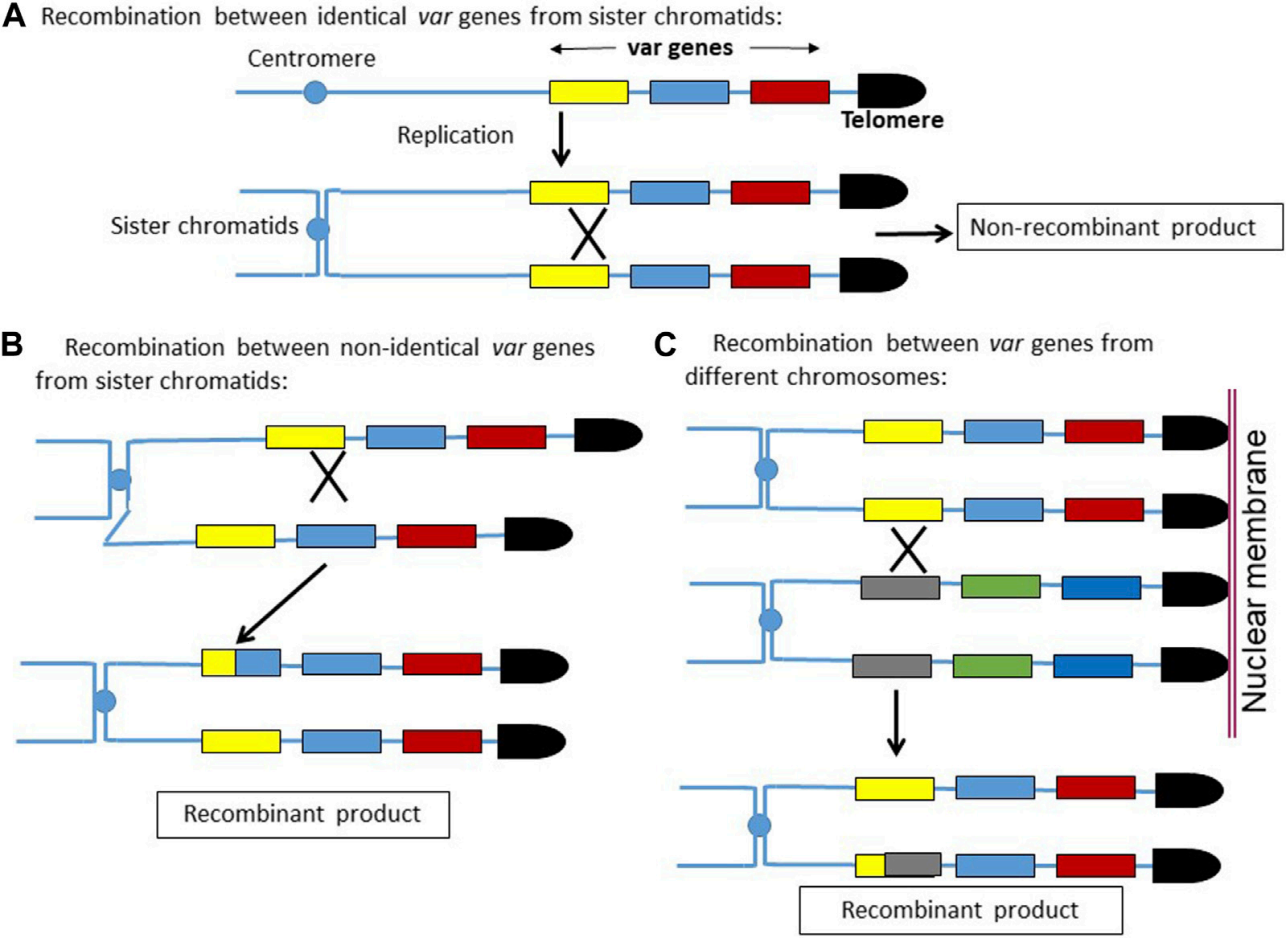
Model depicting how homologous recombinations can create new *var* gene sequences. **(A)** Recombination between two identical var sequences coming from sister chromatids would result in non-recombinant products. **(B,C)** Recombination between two non-identical var sequences coming from sister chromatids or from non-homologous sub-telomeric regions would result in newer *var* sequences.

We observed higher recombination events in the *var* loci compared to the rest of the genome. This is rather intuitive, as in the haploid *P. falciparum* genome, the homologous template for recombination comes only from sister chromatids, whereas for *var* recombination, there are several possible homologous templates. As depicted in the model (Figure 5), any crossover event between the identical *var* genes coming from sister chromatids would not produce recombinant *var* genes. On the other hand, a recombination between two non-identical *var* genes would produce recombinant *var* sequences, even though the two recombining *var* genes are coming from sister chromatids. Moreover, the recombination between two non-identical *var* genes belonging to different chromosomes would also produce recombinant *var* genes. Due to the clustering of multiple telomeres on the nuclear membrane, the juxtaposition of two *var* genes from different chromosomes is feasible, and hence, the recombination between them is also possible.

PfEMP1, encoded by the *var* multigene family, is the most prominent determinant of antigenic variation in *P. falciparum* and, hence, involved in immune evasion. Out of the 60 or so *var* genes, only one is expressed at a given time and the rest are silenced. The rate of switching from one *var* gene expression to another is approximately 2% per generation. Thus, it is expected that after a finite number of generations, the entire *var* repertoire will be expressed, seriously compromising the parasite’s ability to manifest antigenic variations. This does not happen because of recombination events occurring between different *var* genes that give rise to newer *var* sequences and, thus, result in a never-ending repertoire of *var* sequences. Our finding that the PfRad51-mediated HR mechanism is involved in *var* gene recombination implies that blocking the *P. falciparum* HR pathway could be an excellent strategy to restrict *var* gene diversification in *P. falciparum*.

## Data Availability

The raw data supporting the conclusion of this article will be made available by the authors, without undue reservation.
